# Evolution and Optimality of Similar Neural Mechanisms for Perception and Action during Search

**DOI:** 10.1371/journal.pcbi.1000930

**Published:** 2010-09-09

**Authors:** Sheng Zhang, Miguel P. Eckstein

**Affiliations:** Vision and Image Understanding, Department of Psychology, University of California, Santa Barbara, California, United States of America; New York University, United States of America

## Abstract

A prevailing theory proposes that the brain's two visual pathways, the ventral and dorsal, lead to differing visual processing and world representations for conscious perception than those for action. Others have claimed that perception and action share much of their visual processing. But which of these two neural architectures is favored by evolution? Successful visual search is life-critical and here we investigate the evolution and optimality of neural mechanisms mediating perception and eye movement actions for visual search in natural images. We implement an approximation to the ideal Bayesian searcher with two separate processing streams, one controlling the eye movements and the other stream determining the perceptual search decisions. We virtually evolved the neural mechanisms of the searchers' two separate pathways built from linear combinations of primary visual cortex receptive fields (V1) by making the simulated individuals' probability of survival depend on the perceptual accuracy finding targets in cluttered backgrounds. We find that for a variety of targets, backgrounds, and dependence of target detectability on retinal eccentricity, the mechanisms of the searchers' two processing streams converge to similar representations showing that mismatches in the mechanisms for perception and eye movements lead to suboptimal search. Three exceptions which resulted in partial or no convergence were a case of an organism for which the targets are equally detectable across the retina, an organism with sufficient time to foveate all possible target locations, and a strict two-pathway model with no interconnections and differential pre-filtering based on parvocellular and magnocellular lateral geniculate cell properties. Thus, similar neural mechanisms for perception and eye movement actions during search are optimal and should be expected from the effects of natural selection on an organism with limited time to search for food that is not equi-detectable across its retina and interconnected perception and action neural pathways.

## Author Summary

The brain has two processing pathways of visual information, the ventral and dorsal streams. A prevailing theory proposes that this division leads to different world representations for conscious perception than those for actions such as grasping or eye movements. Perceptual tasks such as searching for our car keys in a living room requires the brain to coordinate eye movement actions to point the high resolution center of the eye, the fovea, to regions of interest in the scene to extract information used for a subsequent decision, such as identifying or localizing the keys. Does having different neural representations of the world for eye movement actions and perception have any costs for performance during visual search? We use computer vision algorithms that simulate components of the human visual system with the two separate processing streams and search for simple targets added to thousands of natural images. We simulate the process of evolution to show that the neural mechanisms of the perception and action processing streams co-evolve similar representations of the target suggesting that discrepancies in the neural representations of the world for perception and eye movements lead to lower visual search performance and are not favored by evolution.

## Introduction

**Figure 1.Virtual:**
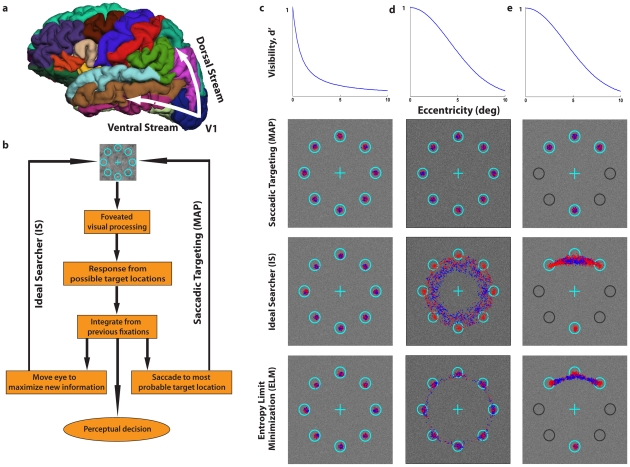
evolution of perception and saccade with different visibility maps, eye movement models and configurations. a. Ventral (perception) and dorsal (action) streams projecting from the primary visual cortex (V1). b. Flow chart for two models of human eye-movement search: Ideal Bayesian Searcher (IS) and the Saccadic targeting model (maximum a posteriori probability model, MAP). c. 8 alternative forced choice target search for steep visibility map. d. 8 alternative forced choice target search for broad visibility map. e. 4 alternative forced choice target search for broad visibility map. Light blue circles outline possible target locations. Location of fixations for 1^st^ (blue) and 2^nd^ saccades (red) for three models: IS, MAP and Entropy Limit Minimization (ELM) in white noise The MAP model simulations include small random saccade endpoint errors to facilitate visualization of the different fixations. Central cross indicates initial fixation point for all models.

Neurophysiology studies of the macaque monkey [1]–[3] support the existence of two functionally distinct neural pathways in the brain mediating the processing of visual information. The behavior of patients with brain damage has led to the proposal that perception is mediated by the ventral stream projecting from the primary visual cortex to the inferior temporal cortex, and that action is mediated by the dorsal stream projecting from the primary visual cortex to the posterior parietal cortex [4]–[6] (Figure 1a). Although there has been debate about whether this separation into ventral/dorsal streams implies that the brain contains two distinct neural representations of the visual world [7]–[12], there has been no formal theoretical analysis about the functional consequences of the two different neural architectures on an animal's survival. Visual search requires animals to move their eyes to point the high-resolution region of the eye, the fovea, to potentially interesting regions of the scene to sub-serve perceptual decisions such as localizing food or a predator. What is the impact of having similar versus different neural mechanisms guiding eye movements and mediating perceptual decisions on visual search performance for an organism with a foveated visual system? We consider two leading computational models of multiple-fixation human visual search, the Bayesian ideal searcher (IS) [13]–[15] and the ideal saccadic targeting model (maximum a posteriori probability, MAP [16], [17]) for a search task of a target in one of eight locations equidistant from initial fixation (Figure 1b). The ideal searcher uses knowledge of how the detectability of a target varies with retinal eccentricity (visibility map) and statistics of the scenes to move the fovea to spatial locations which maximize the accuracy of the perceptual decision at the end of search [13] (Figure 1b). The saccadic targeting model (MAP) makes eye movements to the most probable target location [6], [17] which is optimal if the goal was to saccade to the target rather than collect information to optimize a subsequent perceptual decision [1] (Figure 1b). Depending on the spatial layout of the possible target locations and the visibility map, the IS and MAP strategies lead to similar (Figure 1c) or diverging eye-fixations (Figure 1d–e). For example for a steeply varying visibility map (Figure 1c) both models make eye movements to the possible target locations while for a broader visibility map (Figure 1d–e) the ideal searcher tends to make eye movements in between the possible target locations attempting to obtain simultaneous close-to-fovea processing for more than one location. Covert attention allows both models to select possible target locations and ignore locations that are unlikely to contain the target when deciding on saccade endpoints and making perceptual search decisions [18], [19]. Perceptual target localization decisions for both models are based on visual information collected in parallel over the whole retina, temporally integrated across saccades, and based on the location with highest sensory evidence for the presence of the target. Critically, we implemented the models to have two processing pathways, one determining where to move the fovea and the other stream processing visual information to reach a final perceptual decision about the target location. Rather than having a single linear mechanism or perceptual template (Figure 1b), each pathway in the model had its own neural mechanism which is compared to the incoming visual data at each possible target location. Likelihood ratios [20] of the observed responses for each of the mechanisms under the hypothesis that the target is present or absent at that location are used to make decisions about where to move the eyes and perceptual decisions (see Materials and Methods).

**Figure 2:**
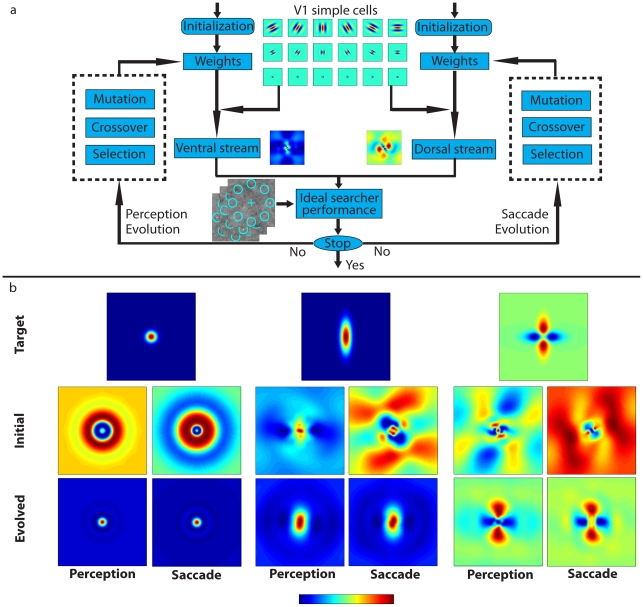
Virtual evolution of two separate streams with the genetic algorithms for three different targets. a. Virtual evolution of the perception (ventral stream) and saccade (dorsal stream) templates constructed from different linear combinations of twenty four different V1 simple cells which spanned the target (Gabor functions with center frequencies, 0.5, 1, 2, 4 cycles/degree for 6 different orientations, 30 degrees apart, and octave bandwidths). Probability of survival of an individual depends on search accuracy of the ideal searcher approximation (ELM model) with the two templates. b. Top row three different targets (from right to left: isotropic Gaussian, vertical elongated Gaussian and the difference of a vertical and horizontal elongated Gaussians) used in different evolution simulations for search in 1/f noise and a steep visibility map (See Figure 1c, left). All targets are luminance grey patterns but are shown in pseudo-color and scaled for each image to maximize the use of the color scale.

We used a genetic algorithm as a method to find near-optimal solutions for perception and action mechanisms but also to simulate the effects of the evolutionary process of natural selection on the neural mechanisms driving saccadic eye movements and perceptual decisions during search. The computational complexity of the ideal Bayesian searcher makes it difficult to virtually evolve the model (see note 1 in Text S2) and thus we used a recently proposed approximation to the ideal searcher that is computationally faster (Entropy Limit Minimization, ELM [15], [21]). The ELM model chooses the fixation location that minimizes the uncertainty of posterior probabilities over the potential target locations. The decision rule can be simplified to choose the fixation location with the maximum sum of likelihood ratios across potential target locations, each weighted by its squared detectability given the fixation location [15]. The ELM model can be shown to approximate the fixation patterns of the ideal searcher [15] and capture the main characteristics of the fixation patterns of the IS for our task and visibility maps (Figure 1c–e; ELM) (see note 2 in Text S2). The process of virtual evolution started with the creation of one thousand simulated individuals with separate linear mechanisms for perception (ventral) and eye movement programming (dorsal; Figure 2a). Each pathway's template for each individual was created from independent random combinations of the receptive fields of twenty four V1 simple cells. Each simulated individual was allowed two eye movements (see note 3 in Text S2) before making a final perceptual search decision about the location of the target. Performance finding the target in one of eight locations for five thousand test-images (one thousand for natural images) was evaluated and the probability of survival of an individual was proportional to its performance accuracy. A new generation was then created from the surviving individuals through the process of reproduction, mutation and cross-over (Figure 2a). The process was repeated for up to 500 generations.

## Results

**Figure 3:**
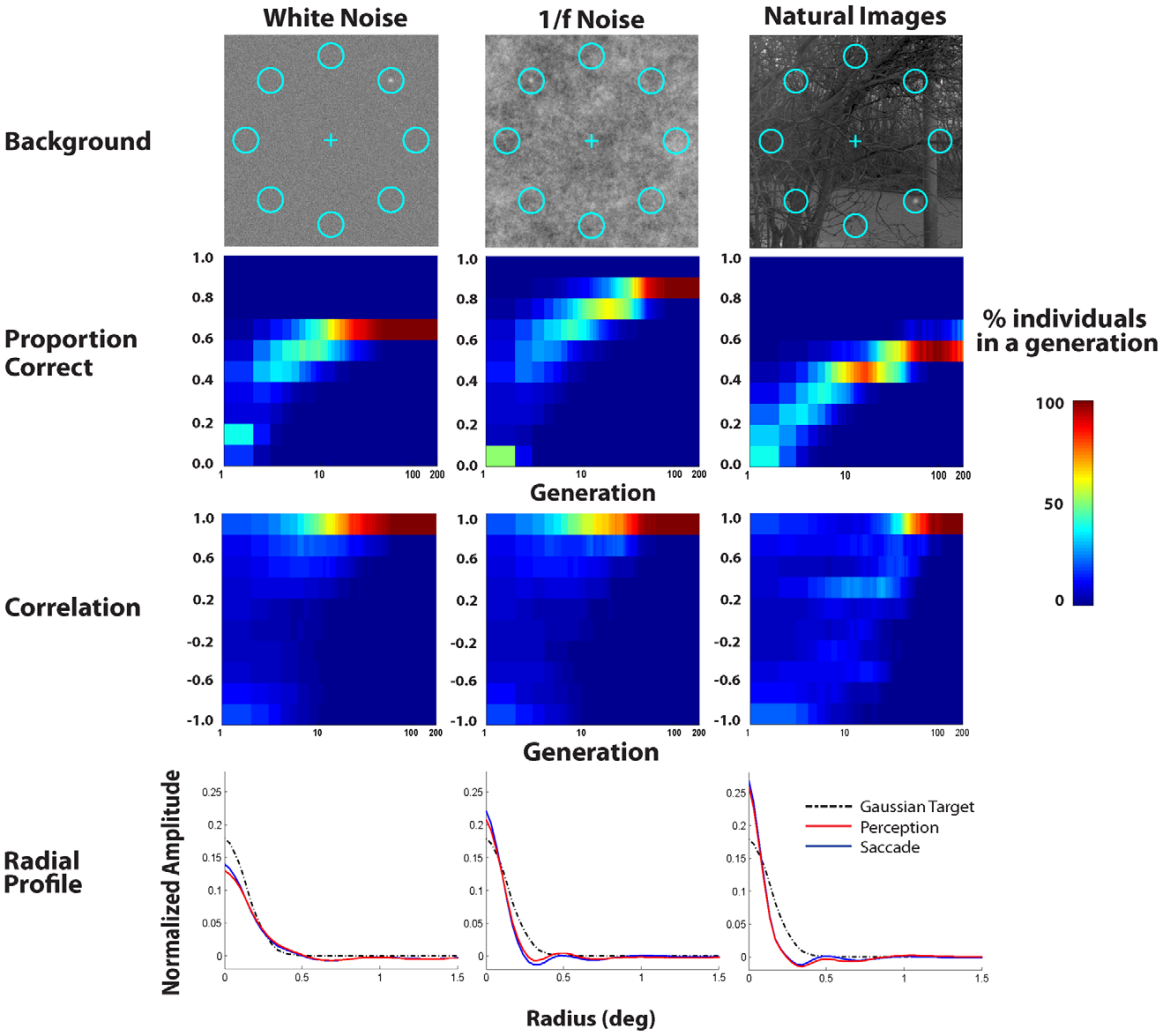
Evolution plots for detecting the isotropic Gaussian target embedded in three different backgrounds. 1^st^ row: Sample images for the 8 alternative forced choice (AFC) search task for an isotropic Gaussian shaped luminance target with a steep visibility map (Figure 1c left) added to white noise, 1/f noise, and natural images. Center of circles indicate the possible target locations and the central cross is the initial fixation position for the models. 2^nd^ row: Distribution of search accuracies for simulated individuals as a function of generation. 3^rd^ row: Distribution of correlations between perception and saccade templates of individuals in each generation. Bottom row: Perception (red) and saccade (blue) templates radial profiles (averaged across all angles) of best performing simulated individual for each background type. Results are averages across ten different virtual evolution runs each with 500 generations. Plots only show data up to the 200^th^ generation for which convergence has occurred. Radial profile of the Gaussian signal is shown in a dashed line for comparison.

We first evolved the ideal searcher approximation (ELM model) for different shape luminance targets (isotropic Gaussian, vertical elongated Gaussian and cross pattern consisting of a positive and negative polarity elongated Gaussian) embedded in 1/f noise and a steep visibility map (Figure 1c). Irrespective of the target shape, virtual evolution led to converging perception (ventral) and saccade (dorsal) mechanisms that are similar to the target (Figure 2b; see Video S1, Video S2, and Video S3 for virtual evolution). To further investigate the generality of the result we evolved the ELM model to search a circular Gaussian target added to backgrounds with different statistical properties: white noise, 1/f noise and importantly, a calibrated set of natural image backgrounds [22]. Figure 3 (2^nd^ row) presents the distribution of perceptual decision accuracies across individuals in a generation and shows that perceptual performances of simulated individuals in the population improve with generations and then converge to an asymptote. We characterized the similarity between the perception and saccade mechanisms by computing the correlations between the 2 dimensional linear mechanisms for each individual in each generation. Figure 3 (3^rd^ row) shows that the distribution of correlations across individuals in the population evolves to unity irrespective of the background type. To visualize in detail the shape of the evolved templates, we analyzed the radial profile of the templates of the highest performing simulated individuals in the last generation (Figure 3; 4^th^ row). For all three backgrounds the saccade and perception templates converge to similar shapes (perception and saccade 2-D template correlations for the best performing templates in the last generation: 0.990±0.006, 0.986±0.013, 0.982±0.013). In addition, the linear mechanisms for the 1/f noise and natural scenes are narrower than those for the white noise and show an inhibitory surround (Figure 3).

**Figure 4:**
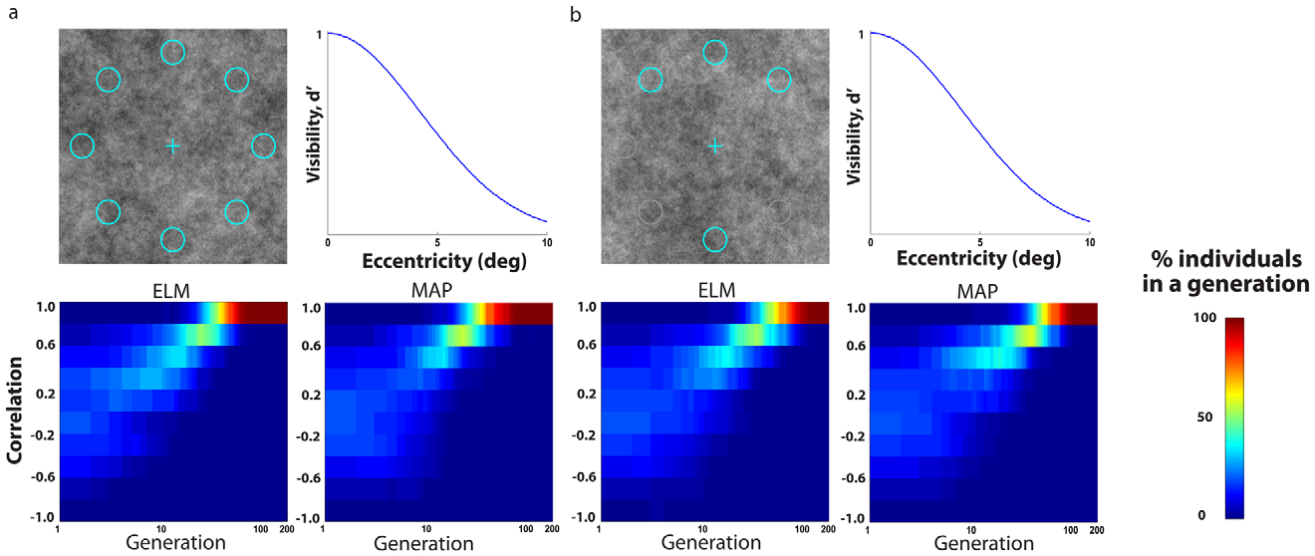
Evolution plots for different models and scenarios detecting the elongated Gaussian target (**Figure 2b**; middle). a. 8 AFC search with a broad visibility map using 1/f noise for the Entropy Limit Minimization model (ELM) and the Saccade Targeting model (MAP); b. 4 AFC with broad visibility map using 1/f noise for the ELM and MAP model. All results based on averages across 10 virtual evolution runs.

These previous results were based on a visibility map that steeply declines with eccentricity and rely on the assumption that humans are near-ideal searchers. We, thus, evolved the mechanisms for the case of a broader visibility map that is similar to that measured for human observers in 1/f noise [15] (Figure 4a) and showed that the convergence of neural mechanisms generalizes to different visibility maps (Figure 4a) and also to a model in which eye movement planning is assumed to follow a saccadic targeting strategy (MAP) rather than approximating an ideal strategy (Figure 4a). Furthermore, Figure 4b shows that there is nothing particular about the symmetry of the eight location configuration search task since similar convergent evolution is observed for an asymmetric four location task (Figure 1e).

**Figure 5:**
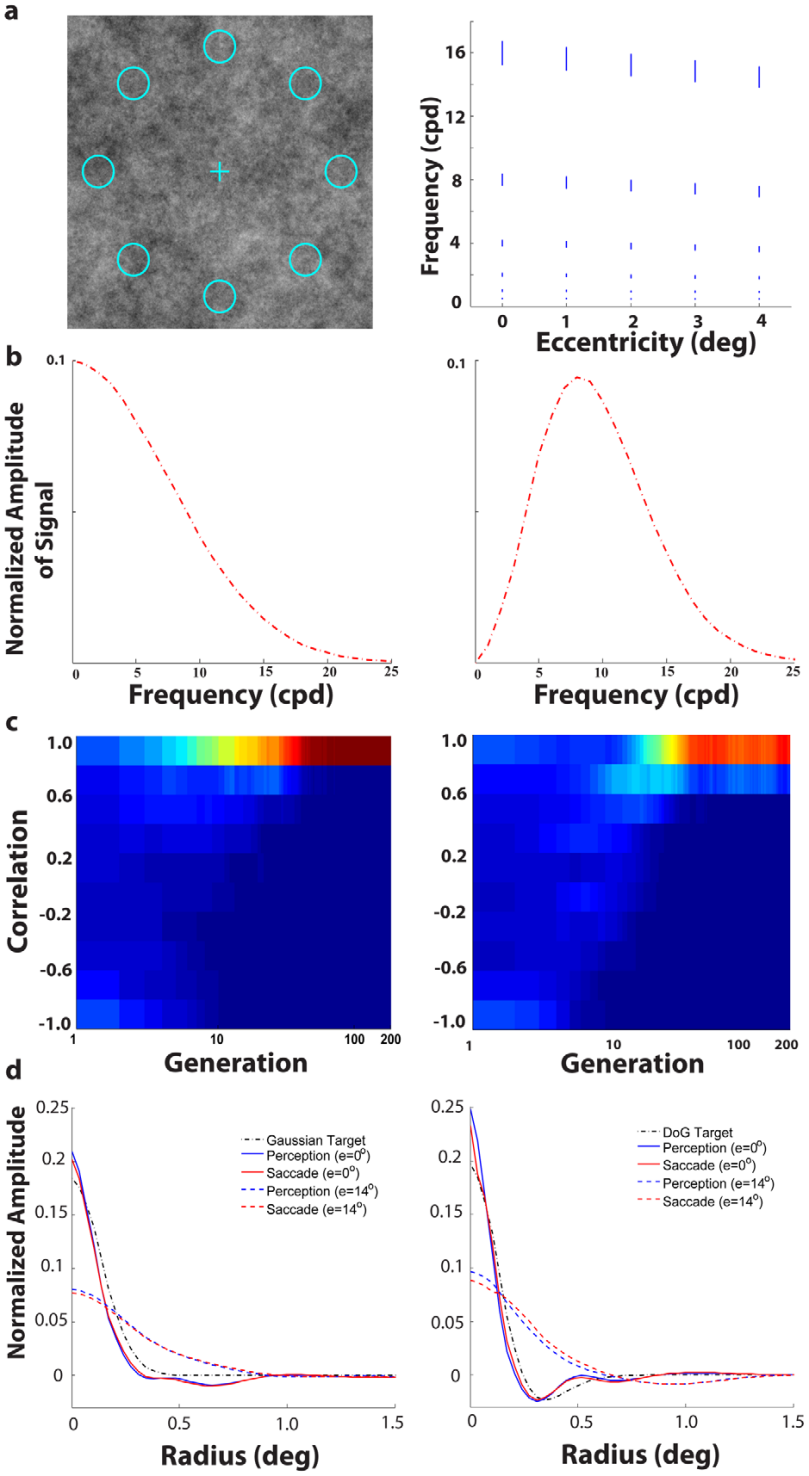
Evolution plots for a model with changing V1 receptive field size/spatial frequency with retinal eccentricity. a. 8 AFC search task in 1/f noise (left) and graph (right) showing the change in central spatial frequency and width of channel in the frequency domain of oriented Gabor functions with retinal eccentricity. b. Radial profiles in the frequency domain of Gaussian target (left) and DoG target (right) with a center frequency of 8 cycles/degree. c. Distribution of correlations between perception and saccade templates of individuals in each generation for Gaussian target (left) and DoG target (right). d. Perception and saccade templates radial profiles (averaged across all angles) of best performing simulated individual for low-frequency Gaussian target (left) and higher frequency DoG target (right).

We also evaluated whether our results would change if the model included the increasing size of V1 receptive fields and lower frequency tuning with retinal eccentricity (see note 4 in Text S2). Figure 5a (right graph) shows the center frequency and bandwidth (standard deviation) of the oriented Gabor receptive fields as a function of retinal eccentricity. The computational time demands of this simulation restricted us to evaluate this model for a fixed set of receptive field weights across eccentricities (see note 5 in Text S2) and limited set of scenarios: 1/f noise, steep visibility map and two targets: a low frequency Gaussian (Figure 5b; left) and a Difference of Gaussians (DoG) with a center frequency of 8 c/deg (Figure 5b; right). Due to the fixed set of weights across eccentricity, in this model the spatial profile of the linear combination of receptive fields scales up with eccentricity. Thus, for each retinal eccentricity category there was a pair of evolved template profiles. Figure 5c shows that convergent evolution still results when receptive field size increases with eccentricity and irrespective of the spatial frequency of the target. Figure 5d presents the similar radial profiles of the of evolved perception and saccade mechanisms for the fovea and a sample peripheral retinal location (perception and saccade 2-D template correlations for the best performing templates in the last generation averaged across retinal eccentricities were: Gaussian target: 0.963±0.008; DoG target: 0.961±0.004).

**Figure 6:**
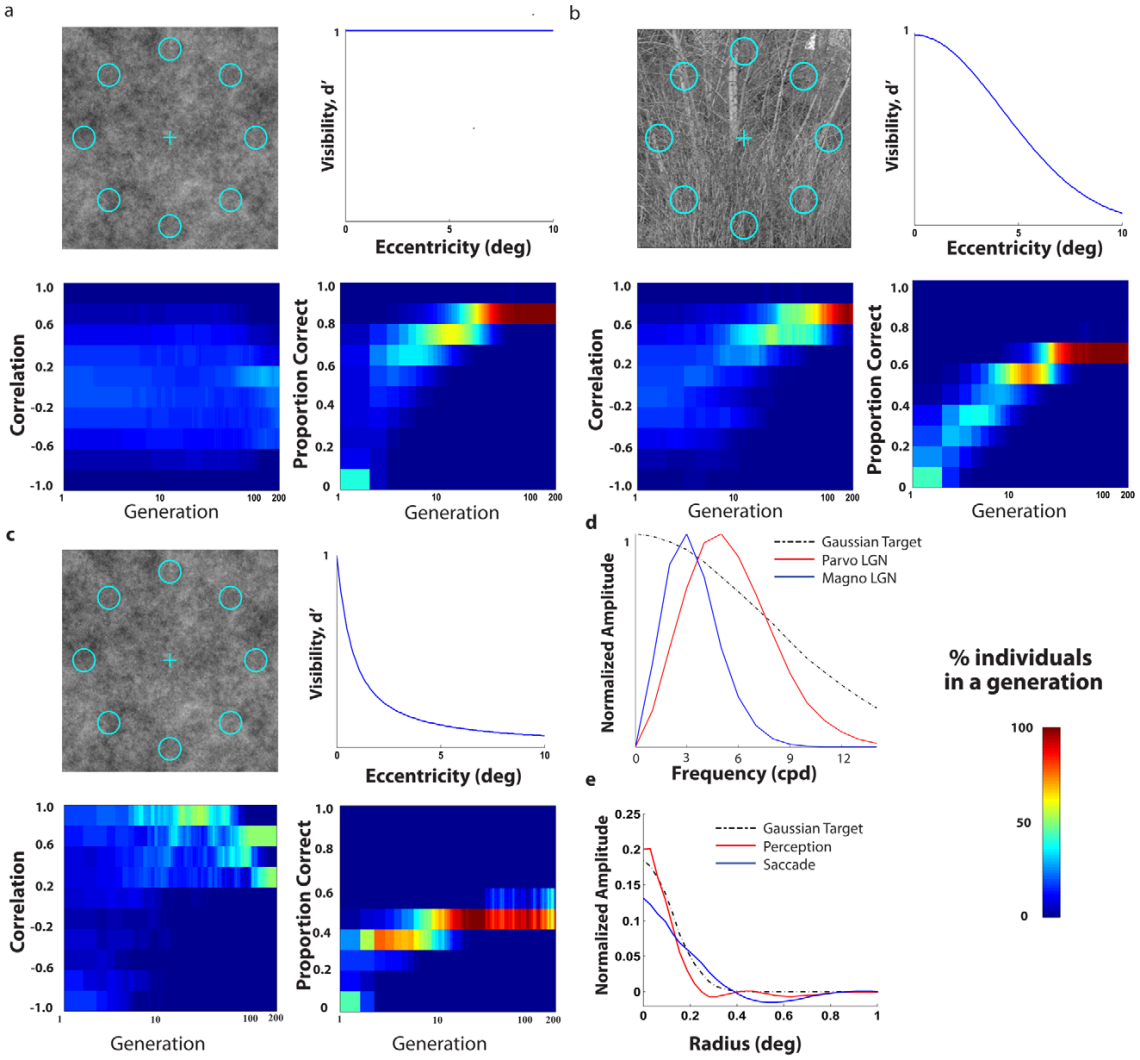
Evolution plots for scenarios which resulted in partial or no convergence of two templates. All proportion correct and correlation plots shows the distribution for individuals in each generation. All results based on averages across 10 virtual evolution runs. a. 8 AFC search of the elongated Gaussian signal for a flat visibility map (ELM model); b. 8 AFC search of the elongated Gaussian signal for a broad visibility map, natural images, but with 8 eye movements which allows the model to fixate on all possible target locations (ELM model); c. 8 AFC search of an isotropic Gaussian signal for a steep visibility map using 1/f noise for the ELM model and considering two visual processing streams with different spatial pre-filtering based on LGN parvocellular and magnocelluar properties; d. Normalized frequency amplitude for Gaussian target, parvocellular LGN cell and magnocellular LGN cell; e. Perception and saccade templates radial profiles (averaged across all angles) of best performing simulated individual for the model with pathway LGN pre-filtering.

Do all scenarios lead to converging evolution of the perception (ventral) and action (dorsal) pathways? No, if we take a case in which the sought target is equally detectable across the retina (flat visibility map), the results show the correlations between the perceptual and saccade templates do not converge to unity (Figure 6a). A second example is a case in which the organism makes a decision after eight eye movements rather than two eye movements (Figure 6b). Because the organism gets to fixate on all eight target locations, there is little added benefit of an efficient saccadic system and the co-evolution is much slower (Figure 6b). A third scenario of partial convergence results if we adopt a strong model of two visual processing streams which spatially pre-filter the visual input based on the properties of the cells in the parvocellular and magnocellular lateral geniculate nucleus (LGN) ([23]; see Figure 6d) and assume no further interaction across pathways. The differential spatial frequency filtering of the two pathways can introduce constraints in the frequency content of the evolved mechanisms preventing a full convergence of the templates (Figure 6e; perception and saccade 2-D template correlations for the best performing templates in the last generation for: 1/f noise: 0.603±0.082). A similar simulation with the same target but white noise instead of 1/f noise also resulted in partial convergence (perception/saccade 2-D template correlation of 0.856±0.046).

## Discussion

We used an approximation to an Ideal Bayesian Searcher (Entropy Limit Minimization model; ELM) to virtually evolve separate linear mechanisms for eye movements and perceptual decisions during visual search for a variety of targets embedded in various synthetic and natural image backgrounds. Evolved templates contain similarities to the target but for the 1/f and natural images they are narrower than the target and contain a subtle inhibitory surrounding not present in the signals but often present in monkey neuronal receptive fields and human behavioral receptive fields [9], [19] (see blue outline in Figure 2b). A previous study has shown that such inhibitory surrounds serve to suppress high amplitude noise in the low frequencies and optimize the detection of spatially compact signals in natural images [24]. The current result extends previous results [24] to show the optimality of inhibitory surrounds during visual search in natural images for an organism with a foveated visual system and saccadic eye movements.

Central to this paper, the mechanisms for perception and saccades evolved to similar representations. This result is robust across different types of backgrounds, signals, visibility maps, and spatial distributions of possible target locations. Due to computational constraints we did not investigate the more general case of allowing the target to appear at any location within the image but there is no particular reason to suggest that our result would differ for this latter general case. In addition, similar convergence between mechanisms was found for what arguably are the most common contender algorithms to model how humans plan eye movements during search: an approximation to the ideal searcher, ELM and a saccadic targeting model; MAP model; [13]. For simplicity our original models did not include receptive fields that increased with retinal eccentricity but an implementation of such a model led to similar convergent evolution for a low and a higher spatial frequency target.

The scenarios for which we did not find full convergent evolution of the linear mechanisms were for cases for which the target was either equi-detectable across the retina or the organism had enough time to fixate all of the possible target locations. Note, however, that for both cases, performance of the evolved individuals does improve with increasing generations (Figure 6a–b) through the evolution of the perceptual template to a target-like structure. Yet, there is no performance advantage for evolving a neural mechanism for saccades that encodes target information because, for these cases different eye movement patterns have little or no impact on perceptual performance. A third scenario which resulted in partial convergence was a two stream model with pathway-specific pre-filtering of the visual input. A strong assumption that there are no interconnections between the two pathways would result in processing constraints based on the early stages of visual processing of both pathways. Inclusion of pre-filtering properties of the parvocellular and magnocellular LGN cells restricted the full convergence of the evolved mechanisms. These finding suggest that if we adopt a strict separation of pathways and take into account properties of LGN cells we should not always expect similar mechanisms driving perception and saccadic decisions during search. The specific circumstances for which we will not find convergent evolution and the degree of similarity between evolved templates will depend on the spatial frequency of the target and background statistical properties (see results for 1/f noise vs. white noise). Yet, is the strict separation of pathways and constraints to the filtering properties of parvocellular (perception) and magnocellular (action) LGN cells tenable for the case of eye movements and perceptual decisions during search? A recent psychophysical study [9] used the same Gaussian target as in the simulations and reverse correlation to show that estimated underlying templates mediating human saccadic actions and perceptual search decisions are similar. Thus, these psychophysical findings would suggest that the strong assumption of no interconnections across pathways and constraints by the early LGN processing might not hold at least for the case of perception and eye movements during visual search.

Together, our present results suggest a theory of why evolution would favor similar neural mechanisms for perception and action during search [9] and provide an explanation for the recent study finding similar estimated underlying templates mediating human saccadic decisions and perceptual decisions. Our findings and theory do not necessarily imply either that one pathway mediates both perception and action nor are they incompatible with the existence of separate magnocellular and parvocellular pathways. Instead, our theory would be consistent with the idea that pathways for perception and oculomotor largely overlap, leading to significant sharing of visual information across pathways [8], [12], [25], [26]. For the case of saccadic eye movements, visual cortical pathways through the frontal eye fields [27] and the lateral intra-parietal cortex [28] play critical roles, as well as brainstem and cortical pathways through the superior colliculus [29]. In addition, studies have related areas in the ventral stream (V4) to target selection of saccades [30], [31]. In addition, the results do not prohibit small differences in visual processing for perception and saccadic action but provide functional constraints on how much discrepancy can exist between neural mechanisms without jeopardizing the survival of the organism.

In the larger context, the similar neural mechanisms for perception and saccade actions should be understood as another effective strategy implemented in the brain, in addition to guidance by target properties [13], [14], [32], [33], optimal saccade planning [15], contextual cues [34], [35] and miniature eye movements [36] to ensure successful visual search. Finally, the approach of the present study demonstrates how the rising field of natural systems analysis [37], [38] can be used in conjunction with virtual evolution and physiological components of the visual system to evaluate whether properties of the human brain might reflect evolved strategies to optimize perceptual decisions and actions that are critical to survival.

## Materials and Methods

### Targets and backgrounds

We assumed a viewing distance of 50cm for the models. Search targets for simulations were: a) A Gaussian target with 0.5539 square root contrast energy (SCE) and a standard deviation of 0.1376 degrees (Figure 1c; 2b left column; 3); b) An elongated Gaussian with 0.9594 SCE, standard deviations of 0.4128 deg. in the vertical direction and 0.1376 degrees in the horizontal direction (Figure 2b center column, Figure 4); c) The difference of a vertically oriented and a horizontally oriented elongated Gaussians with 0.8581 SCE (Figure 2b, right column). The white noise root mean square contrast (rms) was 0.0781. The same rms was used for white noise filtered with the 1/f function (1/f noise). Possible target locations were equidistant 7 degrees from the center fixation cross. Independent external and internal noise samples were refreshed with each saccade for the white and 1/f noise. For the natural images the external backgrounds were fixed but the internal noise refreshed across saccades.

### Model simulations

Here, we briefly describe the models implementations (see Text S1 for detailed mathematical development and details). The initial stage of all three models investigated (ideal searcher, IS; entropy limit minimization, ELM; and saccadic targeting, MAP) is the dot product of a perceptual and saccade template (**w**) with the image data (**g**) at all possible target locations, 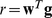 where r is the resulting scalar response and **w** and **g** are expressed as 1-D vectors. The templates for the perceptual decisions and saccade planning were independent and random linear combinations of 24 Gabor functions that spanned the targets: spatial frequencies, 0.5, 1, 2, 4 cycles/degree for 6 different orientations, 30 degrees apart, and with octave bandwidths. A subset of simulations (Figure 6) also modeled pre-processing of the image by separate LGN cells corresponding to the magnocellular (dorsal) and parvocellular (ventral) cells. The filtering was done using DoG functions with different center frequencies (see Text S1 for mathematical details) prior to the processing by the Gabor functions.

Use of a larger number of Gabor functions did not significantly change the evolved templates for the targets considered but required prohibitively longer computational times due to the dimensionality explosion. For the template derived for the case of the isotropic Gaussian target we used an additional constraint of equal weighting for all orientations of the Gabor functions for a given spatial frequency. Most of the simulations used the fixed 24 Gabor functions irrespective of retinal eccentricity. A subset of simulations (see Figure 5) used sets of 24 Gabor functions that increased linearly in size and also decreased in the central frequency tuning with retinal eccentricity (see details in effects of retinal eccentricity section). Template responses were integrated across saccades. Calculation of likelihood ratios use Gaussian probability density functions which depend on the image parameters for the white and 1/f Gaussian noisy images. For the natural images, the likelihood calculation required estimating the probability density function from a training set of 3000 images and fitting the probability density functions with Laplacian distributions convolved with a Gaussian distribution representing the internal noise (see Text S1).

### Effects of retinal eccentricity

Two methods were used to model the detrimental effect of retinal eccentricity on the detectability of the target. The first method which is similar to Najemnik and Geisler [13] was implemented by adding internal noise to the scalar template response: 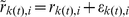, where the additive internal noise scalar value 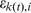 is sampled from a Gaussian distribution which standard deviation (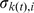) is dependent on the distance ( i.e. retinal eccentricity) between the t^th^ fixation 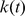 and the template response location *i* out of *m* possible target locations. Also the internal noise was proportional to the template's response standard deviation resulting from the external image variability. The visibility maps referred to as steep and broad (see also Figure S1) were obtained with internal noise standard deviations given by:
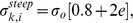
(1a)

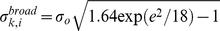
(1b)where σ_o_ is the standard deviation of the template response due to external noise, *e* is the eccentricity in degrees, and the subscripts *k* refer to the fixation location, and *i* to the possible target location. For all models, independent samples of internal noise were used for each saccade and pathways.

The second method to model the effects of retinal eccentricity included internal noise (see above) and also varied the sets of 24 Gabor functions with retinal eccentricity.

The size of Gabor functions increased with the retinal eccentricity (*e*) so that the standard deviation of the spatial Gaussian envelope is given by:
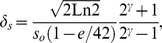
(2)where 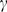 is the bandwidth and 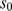 is the center frequency of Gabor function in the fovea. Thus, the standard deviation in the frequency domain of each Gabor function (Figure 5a; right graph) decreases as:
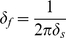
(3)


The center frequency tuning of the Gabor functions (s) linearly decreased with retinal eccentricity: 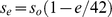.

### The saccadic targeting model

The saccadic targeting or maximum a posteriori probability model (MAP) chooses the location of the next fixation with the maximum product of likelihoods ratios (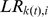) across previous and present fixation (t = 1,…, T):
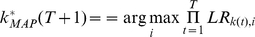
(4)


For the case of white noise and 1/f Gaussian noise the expression can be simplified to the sum of log-likelihood ratios:
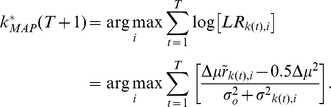
(5)where Δμ is the difference in mean response of the template to the signal plus background and background only and all other symbols are defined above.

### The ideal searcher (IS)

The ideal searcher selects as the next fixation the location that will maximize the probability of finding the target after the eye movement is made:
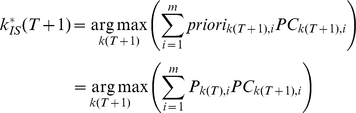
(6)where 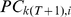 is the proportion correct (PC) given that the target location is *i*, and the next fixation is 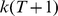. The term 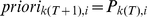 is the prior that the *i* ^th^ location contains the target given the sensory evidence collected up to the present fixation: 
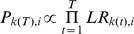
and *m* is the number of possible target locations. For white noise and 1/f noise Gaussian noise, 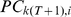 becomes:
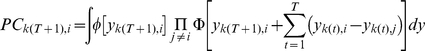
(7)where 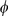 is the probability density function of the Gaussian function in Equation (9a), 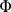 the cumulative density function of the Gaussian function in Equation (9b), 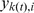
and 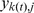, are the log-likelihood ratios which are known scalar values based on acquired visual information,
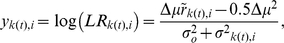
(8a)

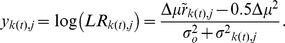
(8b)while 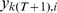 and 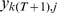 are random variables describing log-likelihoods after the next fixation and described by normal probability density functions:
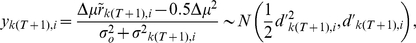
(9a)

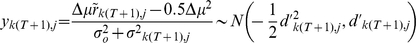
(9b)where 
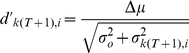
 (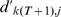) is the detectability at target location *i* (*j*), given fixation at location 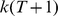. The present formulation is identical to that of Najemink and Gielser [13] but uses likelihood ratios rather than product of posteriors.

### Entropy limit minimization model (ELM model)

The entropy limit minimization model chooses as the next fixation the locations that minimize the expected entropy and can be approximated by maximizing the expected information gain. This can be shown to be approximated by calculating for each potential fixation location, 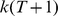, a sum of the posterior probability for each location weighted by the squared detectability given the fixation location [15]:
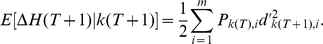
(10)where 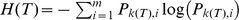 is the Shannon entropy of 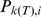, and 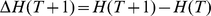 is the information gain.

### Perceptual decisions

For all models, the final perceptual decision about the target location was obtained by combining the likelihood ratios for each possible target locations across all fixations and choosing the location with the highest product of likelihood ratios:
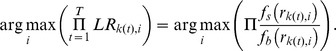
(11)where the likelihoods of the responses given the background only and the target are given 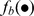 and 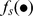 which are the probability density functions (pdf) assumed to be Gaussian (white noise and 1/f noise) or empirically estimated from samples (see next section) for the natural images.

### Natural images

The distribution of template responses for the natural image dataset [22] were estimated from 24,000 image patches extracted from the eight possible target locations for 3000 natural images. We fit the distribution of these responses for each template of each simulated individual with a Laplacian distribution:
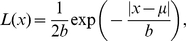
(12)where 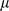 is the mean parameter and 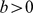 is a scale parameter. To take into account the effect of additive Gaussian internal noise on the probability density function of the template responses we convolved the Laplacian distribution with the Gaussian distributions:
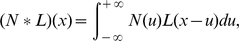
(13)where 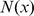 and 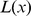 are Gaussian and Laplace probability density functions respectively (see Figure S2).

### Genetic algorithm

We used the Genetic Algorithm Optimization Toolbox (GAOT) [39]. Arithmetic crossover parameter was set to operate 50 times per generation, and uniform mutation to operate 50 times per generation. The selection process used a real-valued roulette wheel selection [38]. A generation consisted of 1,000 individual parameter settings. All individuals were randomly initialized, and allowed to evolve over 500 generations (see Text S1 for additional details). Reported results for each scenario/model were averages across ten simulated evolution runs.

## Supporting Information

Figure S1 Three visibility maps used in present paper.
(0.07 MB TIF)

Figure S2 The probability density function of natural images is estimated from empirical distributions. (a) Gaussian and Laplace distributions fit to the distribution of template responses to natural images. (b) Convolution of Laplacian distribution with a Gaussian internal noise distribution.
(0.15 MB TIF)

Text S1 A detailed description of the methods used in the paper.
(0.39 MB DOC)

Text S2 Some notes for the manuscript.
(0.03 MB DOC)

Video S1 Virtual evolution of linear neural mechanisms (templates) for perception (ventral stream) and saccadic action (dorsal stream) for search of an elongated Gaussian target. Video shows for each generation a perception and saccade template of a randomly sampled simulated individual. Legend below video indicates the generation number.
(7.83 MB GIF)

Video S2 Virtual evolution of linear neural mechanisms (templates) for perception (ventral stream) and saccadic action (dorsal stream) for search of a cross pattern consisting of a positive and negative polarity elongated Gaussian. Video shows for each generation a perception and saccade template of a randomly sampled simulated individual. Legend below video indicates the generation number.
(8.65 MB GIF)

Video S3 Virtual evolution of linear neural mechanisms (templates) for perception (ventral stream) and saccadic action (dorsal stream) for search of an elongated Gaussian target in natural images. Video compares a pair of evolved perception and saccade templates and a pair of randomly generated templates.
(3.71 MB GIF)
