# Supplementary figures and images for: Evolution and Optimality of Similar Neural Mechanisms for Perception and Action during Search
Source: PLoS Comput Biol. 2010 Sep 9;6(9):e1000930. doi: 10.1371/journal.pcbi.1000930 (PMC2936525; doi:10.1371/journal.pcbi.1000930)

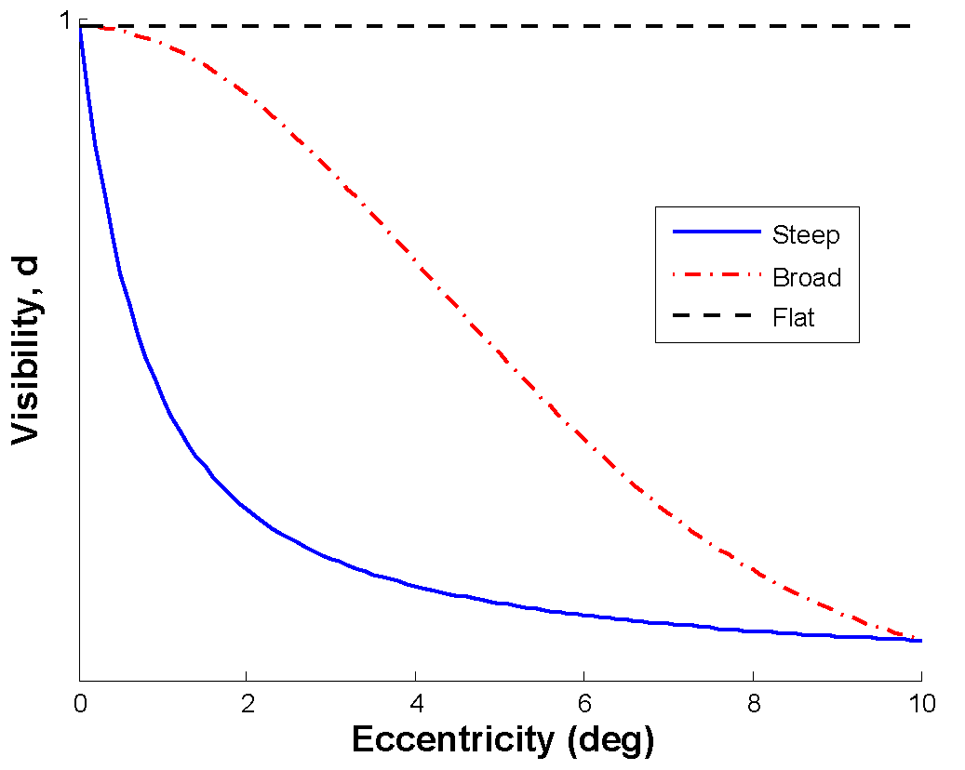

Supplement: Figure S1 — Three visibility maps used in present paper. (0.07 MB TIF) [file pcbi.1000930.s001.tif]

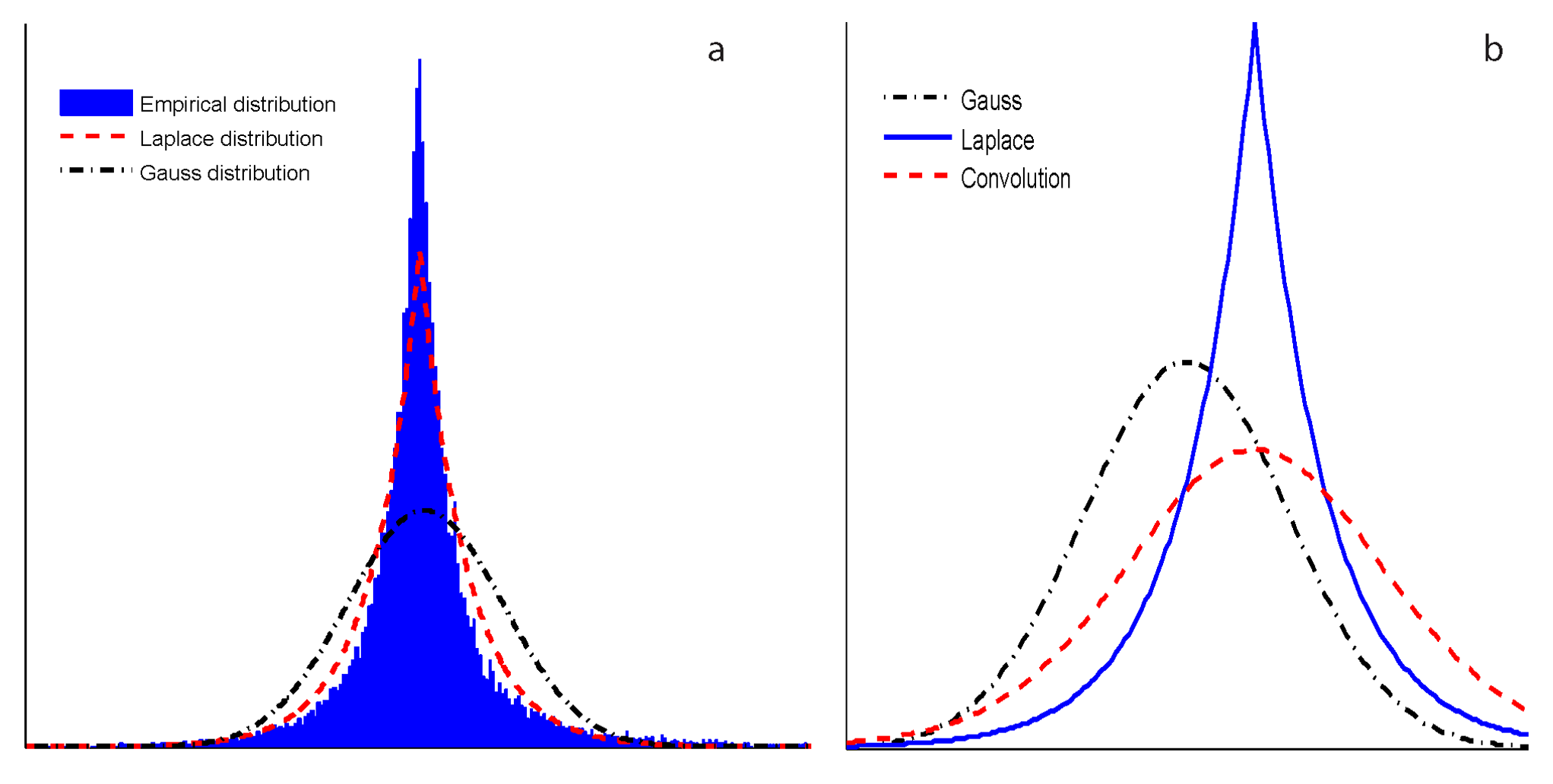

Supplement: Figure S2 — The probability density function of natural images is estimated from empirical distributions. (a) Gaussian and Laplace distributions fit to the distribution of template responses to natural images. (b) Convolution of Laplacian distribution with a Gaussian internal noise distribution. (0.15 MB TIF) [file pcbi.1000930.s002.tif]

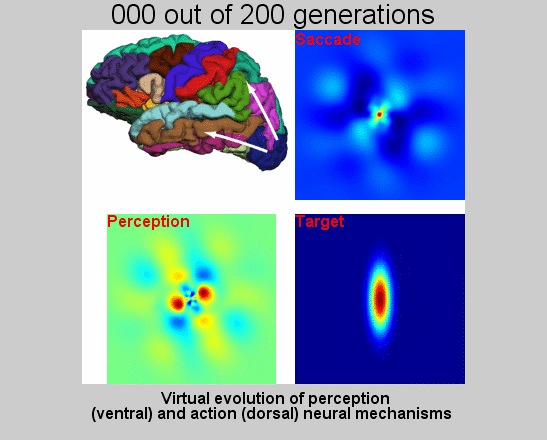

Supplement: Video S1 — Virtual evolution of linear neural mechanisms (templates) for perception (ventral stream) and saccadic action (dorsal stream) for search of an elongated Gaussian target. Video shows for each generation a perception and saccade template of a randomly sampled simulated individual. Legend below video indicates the generation number. (7.83 MB GIF) [file pcbi.1000930.s005.gif]

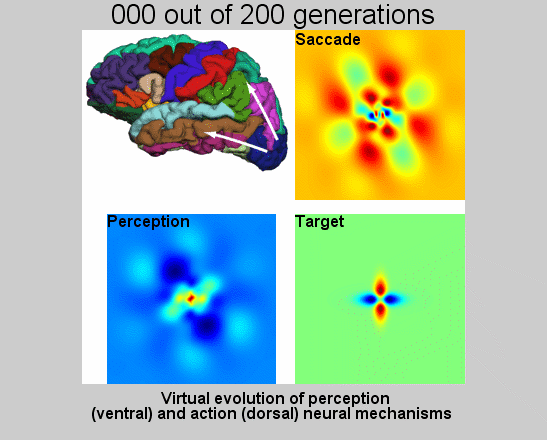

Supplement: Video S2 — Virtual evolution of linear neural mechanisms (templates) for perception (ventral stream) and saccadic action (dorsal stream) for search of a cross pattern consisting of a positive and negative polarity elongated Gaussian. Video shows for each generation a perception and saccade template of a randomly sampled simulated individual. Legend below video indicates the generation number. (8.65 MB GIF) [file pcbi.1000930.s006.gif]

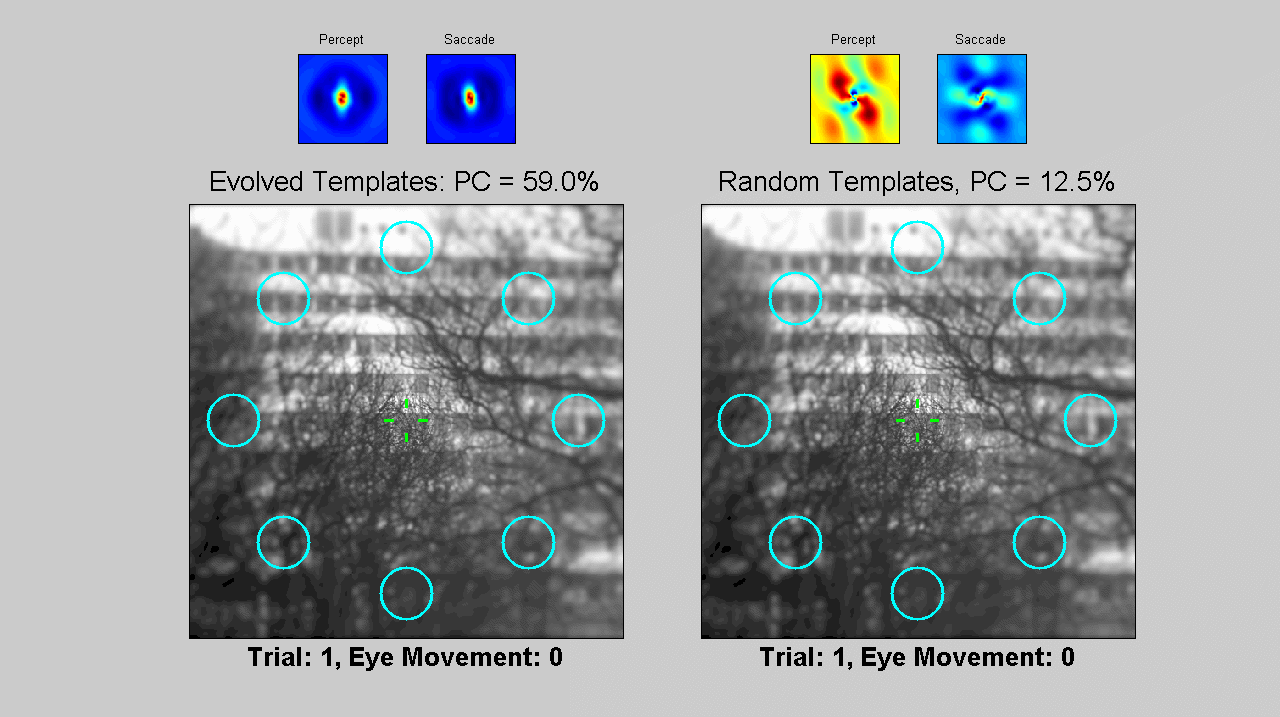

Supplement: Video S3 — Virtual evolution of linear neural mechanisms (templates) for perception (ventral stream) and saccadic action (dorsal stream) for search of an elongated Gaussian target in natural images. Video compares a pair of evolved perception and saccade templates and a pair of randomly generated templates. (3.71 MB GIF) [file pcbi.1000930.s007.gif]
