# Supplementary material for: Evolution and Optimality of Similar Neural Mechanisms for Perception and Action during Search
Source: PLoS Comput Biol. 2010 Sep 9;6(9):e1000930. doi: 10.1371/journal.pcbi.1000930 (PMC2936525; doi:10.1371/journal.pcbi.1000930)
Supplement: Text S1 — A detailed description of the methods used in the paper. (0.39 MB DOC) [file pcbi.1000930.s003.doc]

**Supplementary Methods**

Here, we detail the mathematical expressions for the ideal searcher (IS), the saccadic targeting model (maximum a posteriori probability model; MAP) and entropy limit minimization (ELM), the visibility maps, the set of Gabor functions which were linearly combined to construct the perception and saccade templates, implementation of separate visual streams, estimation of probability density functions for natural images and the genetic algorithm. The supplementary materials are organized in the following sections: 1) Initial linear stage of models and decision variables; 2) Templates as linear combinations of channels; 3) Visibility maps for a foveated visual system; 4) Computational models of multiple fixation visual search; 5) Implementation of models with two separate processing streams for perception and eye movements; 6) Estimation of probability density functions of template responses for natural images; 7) Genetic algorithms methods.

1. **Initial Linear Stage of models and decision variables**

All three multiple fixation models (MAP, ELM and IS) start with an initial linear operation common to most models based statistical decision theory for forced choice localization tasks in noise [1-4]. Let the vector$\mathbf{g}$denote the grey values of the pixels in a 2-D image patch. We refer to a signal-present patch of the image as $\mathbf{g}_{0}^{+}$ and to a signal-absent patch as $\mathbf{g}_{i}^{-}$, $i=1,\text{ }2,\text{ }\cdots M-1$, in an *M* alternative forced choice (MAFC) task. In a forced choice trial, a signal, $\mathbf{s}$, is added to the image background, $\mathbf{b}_{0}$, in one of M possible locations chosen at random: $\mathbf{g}_{0}^{+}=\mathbf{b}_{0}+\mathbf{s}$. For target-absent locations, the image patch consists of: $\mathbf{g}_{i}^{-}=\mathbf{b}_{i}$. Note that $\mathbf{s}$ is a deterministic vector, and image backgrounds are considered to be random with some statistical properties.

The linear model observer forms a response variable,$r$, as an inner product between $\mathbf{g}$ and a vector of weights $\mathbf{w}$: $r=\mathbf{w}^{T}\mathbf{g}$. We will refer to the set of weights applied by the model to the image as the template. When presented with $\mathbf{g}_{0}^{+}$ and $\mathbf{g}_{i}^{-}$, the observer forms M response variables $r_{0}^{+}$ and M-1 $r_{i}^{-}$. An optimal rule to make decisions in a multiple forced choice task is to compare for each location the posterior probability that the target is at that location given the responses at all M locations $P(H_{i}\left|r_{1},...r_{m}\right.)$ and choose the location with the highest posterior probability. The posterior probability is related to the likelihood by Bayes’ rule:

$P(H_{i}\left|r_{1},...,r_{m}\right.)=\frac{P(r_{1},...,r_{m}\left|H_{i}\right.)P(H_{i})}{P(r_{1},...,r_{m})}$ (S1)

where $H_{i}$ is the *i*^th^ hypothesis which refers for our case is the target being at the *i*^th^ location. $P(H_{i})$ is the prior probability of the target being at the *i*^th^ location and $P(r_{1},...,r_{m}\left|H_{i}\right.)$ is the likelihood of the template responses at the m locations $(r_{1},...,r_{m})$ given the *i*^th^ hypothesis. One can equivalently make optimal decisions using the ratio of likelihood (LR) of the response $r_{i}$ given target present or absence at the *i*^th^ location weighted by *i*^th^ location’s prior probability of containing the target ($\pi_{i})$:

$P(H_{i}\left|r_{1},...,r_{m}\right.)\propto\pi_{i}LR_{i}=\pi_{i}\frac{f_{s}(r_{i})}{f_{b}(r_{i})}$ (S2)

where the likelihoods of the responses given the background only and the target are given $f_{b}(\bullet)$ and $f_{s}(\bullet)$ which are the probability density functions (pdf). Assuming equal prior probabilities, the observer makes a correct decision if $LR_{0}>\max\left(LR_{i}\right)$, and an incorrect decision otherwise. This is the decision-making strategy for traditional single fixation search (or a non-foveated model) with noise that is fixed through time.

If the noise (external or internal to the observer) is time-variant and independent through time, we define the dynamic posterior as:

$P_{k(T),i}=\prod_{t=1}^{T} P(H_{i}\left|r_{k(t),1},...,r_{k(t),m}\right.)$, (S3)

where $r_{k(t),i}$ is the template response at potential location *i* for fixation $k(t)$. Alternatively one can make decisions with the product of likelihood ratios:

$P_{k(T),i}\propto\prod_{t=1}^{T} \pi_{i}LR_{k(t),i}=\prod\pi_{i}\frac{f_{s}\left(r_{k(t),i}\right)}{f_{b}\left(r_{k(t),i}\right)}$ (S4)

1. **Templates as linear combination of channels**

We considered templates that are constructed from a linear combination of a set of pre-processing linear channels. We describe a set of channels with a matrix$\mathbf{V}\in R^{D\text{ }xC}$, where each column represents a 2-D channel arranged as a column vector. The model operates on the output of the channels $\mathbf{V}^{T}\mathbf{g}$ instead of the image itself. The decision variable $r$ can be rewritten as: $r=\mathbf{a}^{T}\mathbf{V}^{T}\mathbf{g}$, where $\mathbf{a}\in R^{C}$ is a vector of channel weights. The effective template $\mathbf{w}$is a linear combination of the channels weighted by $\mathbf{a}$, $\mathbf{w}=\mathbf{Va}$.

In the context of a linear channel model, the goal becomes to find the optimal channel weights$\mathbf{a}$.

This pre-processing of the image into channel outputs significantly reduces the spatial complexity because the number of channels is much smaller than the dimensionality of image patches. We applied a subset of Gabor functions that model the spatial frequency and orientation tuning of simple cells in the primary visual cortex [5]. The Gabor functions proposed by Marcelja [6] and Daugman [7] are parameterized by$\sigma_{x}$ and $\sigma_{y}$, the standard deviations of the Gaussian envelope in the x and y directions, *s*, the spatial frequency in cycles per degree, $\phi$, the preferred spatial phase, and $\theta$, the orientation. The Gabor function is mathematically expressed as , (S5)

Where$x'=x\cos\theta+y\sin\theta$ and $y'=-x\sin\theta+y\cos\theta$. In our study, $\sigma_{x}=\sigma_{y}$, $\phi=0$, and $\theta$ varies from 0˚ to $180^{\circ}$ in steps of $30^{\circ}$. Thus, we only use a subset of functions centered on the possible target locations and with even phase. Note that $\sigma_{x}$ and *s* determine the spatial frequency bandwidth$\gamma$, which is the width (full width at half max) of the spatial frequency tuning curve measured in octaves. More precisely,

$\sigma_{s}=\frac{\sqrt{2\text{Ln}2}}{s}\frac{2^{\gamma}+1}{2^{\gamma}-1}$. (S6)

Based on previous research [8], we set the bandwidth to$\gamma=1$. Four spatial frequencies were used, *s* = 0.5, 1, 2, 4 cycles/degree, with width parameter set according to Equation (S6). A total of 4 spatial frequencies (*s*) and 6 orientations ($\theta$) result in a total of 24 Gabor channels. A full model would use 8 spatial frequencies, 8 orientations and 2 phases (even and odd phase) resulting in a total of 128 channels. Because most of our targets were low frequency we reduced the dimensionality of the problem by using a subset of channels that can span the target.

1. **The Visibility Map for a foveated visual system**

The detrimental effect of retinal eccentricity on the detectability of the target was implemented by adding internal noise to the scalar template response: $\tilde{r}_{k(t),i}=r_{k(t),i}+\varepsilon_{k(t),i}$, where the additive internal noise scalar value$\varepsilon_{k(t),i}$ is sampled from a Gaussian distribution which standard deviation ($\sigma_{k(t),i}$) is dependent on the distance between the fixation $k(t)$ and the template response location, *i* (i.e. retinal eccentricity). Also, the internal noise was proportional the template’s response standard deviation resulting from the external image variability [9].

The models’ internal responses to the target relative to the inherent noise can be quantified (prior to likelihood calculations) using $d'$, detectability index, which is the

average difference ($\Delta\mu$) between the template response to the background plus target and background only, divided by the standard deviation of the template response due to external ($\sigma_{o}$) and internal noise ($\sigma_{k,i})$:

$d_{k,i}^{'}=\frac{\Delta\mu}{\sqrt{\sigma_{o}^{2}+\sigma_{k,i}^{2}}}$ (S7)

where *k* is the fixation location, and *i* is a template response location in the image patch. The functions that determined the index of detectability at each eccentricity (distance in visual degrees between the fixation *k* and the location *i*) are plotted in Figure S1. As show in Figure S1, in this paper, we use two foveated visibility maps (steep and broad) and a third flat visibility map:

$\sigma_{k,i}^{steep}=\sigma_{o}\left[0.8+2e\right]$, (S8a)

$\sigma_{k,i}^{broad}=\sigma_{o}\sqrt{1.64\exp\left(e^{2}/18\right)-1}$ , (S8b)

$\sigma_{k,i}^{flat}=\sigma_{o}$ (S8c)

1. **Computational models of multiple fixation visual search**

Models are derived for the case in which the internal noise samples are independent with each fixation for all backgrounds. In addition the external noise is also independently sampled with fixation for the white and 1/f noise.

**4.1. Decision rules for eye movements**

**4.1.a. Saccade Targeting (MAP) Model**

Given the template response $r_{k(t),i}$ across different location i and across eye movements t, the MAP model selects for the next fixation the location with the highest posterior$P_{k(T),i}$.

$$k_{MAP}^{*}(T+1)=\mathop{\mathrm{argmax}}\limits_{i} P_{k(T),i}$$

$$=\mathop{\mathrm{argmax}}\limits_{i} \prod_{t=1}^{T} LR_{k(t),i}$$

$=\mathop{\mathrm{argmax}}\limits_{i} \sum_{t=1}^{T} \log[LR_{k(t),i}]$. (S9)

where we assumed the priors to be equal across locations. To avoid overflow, the sum of log likelihood ratios is used instead of the product of likelihood ratios, which is a monotonic transformation and will produce the exactly same result.

We now take a Gaussian distribution as an example to show how to use MAP model for practical simulations. Suppose the probability density function (pdf) of backgrounds is normal, i. e. Gaussian with zero mean and unit standard deviation (i.e.$\delta=1$). Notice that one can always transform any Gaussian distribution to be normal by translating and scaling. Since the signal is known and additive, the pdf of signal plus background is also Gaussian with mean $\mu$ and unit standard deviation. That is,

(S10a)

. (S10b)

Thus, the log likelihood ratio is

$$\log[LR_{k(t),i}]=\log\left[\frac{f_{s}(r_{k(t),i})}{f_{b}(r_{k(t),i})}\right]$$

$=\log\left[\frac{\frac{1}{\sqrt{2\pi}}\exp\left(-\frac{(r_{k(t),i}-\mu)^{2}}{2}\right)}{\frac{1}{\sqrt{2\pi}}\exp\left(-\frac{r_{k(t),i}^{2}}{2}\right)}\right]=\mu r_{k(t),i}-\frac{1}{2}\mu^{2}$ (S11)

Based on the Gaussian assumption, the MAP model determines the next fixation by summing the template response and then selecting the maximum.

$$k_{MAP}^{*}(T+1)=\mathop{\mathrm{argmax}}\limits_{i} \sum_{t=1}^{T} \log\left[LR_{k(t),i}\right]$$

$=\mathop{\mathrm{argmax}}\limits_{i} \mu\sum_{t=1}^{T} r_{k(t),i}$. (S12)

Note that the constant component ($\frac{1}{2}\mu^{2}$) in Equation (S11) has been safely dropped. Equation (S12) only considers the template response without internal noise. When the internal noise is present, we have to take account of the statistical properties of the internal noise and its dependence on eccentricity when determining the next fixation. If the distribution of internal noise is characterized as$\varepsilon_{k(t),i}\sim N(0,\text{ }\sigma_{\text{k(t),}i})$, then the distributions of the perturbed template response are

(S13a)

(S13b)

Thus, the log likelihood ratio is modified to become:

. (S14)

Accordingly, by considering the internal noise as well as the external noise (i.e. the background), the MAP model determines the next fixation by summing the weighted template responses:

$$k_{MAP}^{*}(T+1)=\mathop{\mathrm{argmax}}\limits_{i} \sum_{t=1}^{T} \log\left[LR_{k(t),i}\right]$$

. (S15)

The MAP model maximizes the probability of finding the target based on the information collected up to present$t=T$.

**4.1.b. Ideal Searcher (IS) Model**

The ideal searcher [10] considers one step further than the saccadic targeting (MAP) model and selects for the next fixation the location that will maximize the expected proportion correct of the perceptual decision after the eye movement is executed:

$$k_{IS}^{*}(T+1)=\mathop{\mathrm{argmax}}\limits_{k(T+1)} \left(\sum_{i=1}^{m} priori_{k(T+1),i}PC_{k(T+1),i}\right)$$

$\mathop{=\mathrm{argmax}}\limits_{k(T+1)} \left(\sum_{i=1}^{m} P_{k(T),i}PC_{k(T+1),i}\right)$ (S16)

where $PC_{k(T+1),i}$ is the proportion correct (PC) given that the target location is *i*, and the next fixation is $\text{k(T}+\text{1)}$. A difference between the IS and MAP model (Equation S12 vs. Equation S16) is that the fixations of the IS model are not limited to the potential target locations. Equation (S16) can be conceptualized as the sum of expected proportion correct weighted by present posteriors for each location. PC is equal to the probability that posterior probability at the true target location i will be greater than that at any other location:

$PC_{k(T+1),i} = \text{Prob}\left(P_{k(T+1),i}\geq P_{k(T+1),1},\cdots,P_{k(T+1),i}\geq P_{k(T+1),m} | H_{i}\right)$. (S17)

The goal now is to compute the proportion correct analytically. Based on Equation (S4) and assuming equal priors for all locations, then:

$P_{k(T+1),i}\propto\prod_{t=1}^{T+1} LR_{k(t),i}$ (S18)

Assuming conditional independence, Equation (S17) can be rewritten as the product of probabilities.

$$PC_{k(T+1),i} = \prod_{j\neq i} \text{Prob}\left(P_{k(T+1),i}\geq P_{k(T+1),j} | H_{i}\right)$$

$$= \prod_{j\neq i} \text{Prob}\left(\prod_{t=1}^{T+1} LR_{k(t),i}\geq\prod_{t=1}^{T+1} LR_{k(t),j} | H_{i}\right)$$

$$= \prod_{j\neq i} \text{Prob}\left(\frac{\prod_{t=1}^{T} LR_{k(t),i}LR_{k(T+1),i}}{\prod_{t=1}^{T} LR_{k(t),j}LR_{k(T+1),j}}\geq1 | H_{i}\right)$$

$$= \prod_{j\neq i} \text{Prob}\left(\sum_{t=1}^{T} \log\left(\frac{LR_{k(t),i}}{LR_{k(t),j}}\right)+\log\left(\frac{LR_{k(T+1),i}}{LR_{k(T+1),j}}\right)\geq0 | H_{i}\right)$$

$= \prod_{j\neq i} \text{Prob}\left(\sum_{t=1}^{T} \left[y_{k(t),i}-y_{k(t),j}\right]+y_{k(T+1),i}-y_{k(T+1),j}\geq0 | H_{i}\right)$, (S19)

where

, (S20a)

. (S20b)

Note that when $t\leq T$, $y_{k(t),i}$and $y_{k(t),j}$, the log-likelihood ratios are known scalar values based on acquired visual information, but $y_{k(T+1),i}$and $y_{k(T+1),j}$ are random variables describing log-likelihoods after the next fixation and are only known statistically . Considering the effects of internal noise as in Equations (S13a) and (S13b)

(S21a)

. (S21b)

Thus the log-likelihood ratios, $y_{k(T+1),i}$ and $y_{k(T+1),j}$ are also Gaussian[[1]](#footnote-2)

(S22a)

(S22b)

where $d'_{k(T+1),i}=\frac{\Delta\mu}{\sqrt{\sigma_{o}^{2}+\sigma_{k(T+1),i}^{2}}}$ is the detectability at target location i, given fixation at location $k(T+1)$. Based on Equation (S22a) and Equation (S22b), we make two observations. First, at the target location i, the mean of the log-likelihood ratio ($y_{k(T+1),i}$) is positive and at any other location j, the mean log-likelihood ratio is negative. Second, both the means and standard deviations are related to the detectability, which depends on the eccentricity, the distance from fixation to the location. In particular, when the detectability $d'_{k(T+1),i}$ decreases, the distributions for both $y_{k(T+1),i}$ and $y_{k(T+1),j}$ move toward zero and become narrower; when the detectability $d'_{k(T+1),i}$ increases, the distributions of$y_{k(T+1),i}$ and $y_{k(T+1),j}$ move apart and become broader.

Proportion correct in Equation (S19) can be rewritten as

$PC_{k(T+1),i}= \int\varphi\left[y_{k(T+1),i}\right]\prod_{j\neq i} \Phi\left[y_{k(T+1),i}+\sum_{t=1}^{T} \left(y_{k(t),i}-y_{k(t),j}\right)\right]dy$ (S23)

where $\varphi$ is the normal probability density function in Equation (S22a) and $\Phi$ is the normal cumulative density function (cdf) in Equation (S22b).

For natural images, we estimate the pdf from empirical distributions. For detailed description, please refer to Supplementary Section (6).

**4.1.c.** **Entropy Limit Minimization (ELM) Model**

The IS model is optimal in terms of the maximum probability of finding the target. However, it may be unlikely that a biological visual system would implement the IS model because of its complexity. An efficient but near-optimal model [11] has been proposed based on maximizing the expected information gain. Rather than maximizing the probability of finding the target, their new criterion for selecting the next fixation is to minimize the expected entropy of the distribution of the posterior probabilities across the potential target locations. More specifically, the information theoretic model will select fixations that are expected to result in the smallest entropy of the posterior probabilities over the potential location after the fixation is made.

$k_{ELM}^{*}(T+1)=\mathop{\mathrm{argmin}}\limits_{k(T+1)} E\left[H(T+1)|k(T+1)\right]$, (S24)

where $H(T)=-\sum_{i=1}^{m} P_{k(T),i}\log\left(P_{k(T),i}\right)$ is the Shannon entropy of $P_{k(T),i}$. Based on certain assumptions, Najemnik and Geisler [11] have shown that minimizing the expected entropy can be approximated by maximizing the expected information gain:

. (S25)

Thus, selecting the fixation that maximizes the expected information gain is equivalent to the weighted sum of the current posteriors across the potential target locations and then selecting the maximum.

**4.2. Perceptual search decision after multiple fixations**

For all models, the final perceptual decision about the target location was obtained by combining the likelihood ratios for each possible target locations across all fixations and choosing the location with the highest product of likelihood ratios:

$\mathop{\mathrm{argmax}}\limits_{i} \left(\prod_{t=1}^{T} LR_{k(t),i}\right)=\mathop{\mathrm{argmax}}\limits_{i} \left(\prod\frac{f_{s}\left(r_{k(t),i}\right)}{f_{b}\left(r_{k(t),i}\right)}\right)$. (S26)

Proportion correct of the perceptual decision in localizing the target was calculated for each model by the tallying the outcome of the model’s perceptual decisions over a finite set of trials (3,000-5,000).

1. **Implementation of models with two separate processing streams for perception and eye movements**

**5.1 Two processing streams with no differential pre-filtering**

Section 4 detailed the decision rules used to make eye movements and the perceptual decisions using the response variables obtained from templates. Our current interest is to investigate the relationship between the templates mediating perceptual and eye movement decisions. Thus, for all visual search model (MAP, IS, ELM) the deployment of eye movements and the final perceptual decisions were mediated by two independent templates. Each template was a linear combination of Gabor functions representing V1 simple cells. We use two separate templates and set of channel weights ($\mathbf{a}_{p}$and$\mathbf{a}_{s}$) to represent templates used for perceptual decision and saccade selection. As in Equation (S2), the perception decision is made based on the product of likelihood ratios, which is derived from the response of the perception template $\mathbf{a}_{p}$. As for saccade selection, we have three models (MAP, IS, ELM), each of which is derived from the response of the saccade template $\mathbf{a}_{s}$. Thus the responses and probability density functions used to calculate likelihoods for the saccade and perception decisions were different.

- 1. **Two processing streams with no interconnections and LGN cells with different spatial frequency pre-filtering**

Physiological studies [12] have shown that the two neural pathways have distinct associated cells in the lateral geniculate nucleus (LGN) with different spatial frequency responses. The parvo (ventral pathway) cells have smaller receptive fields, while the magno (dorsal pathway) cells have larger receptive fields. In general, the receptive fields of LGN cells can be modeled by using difference of Gaussian (DoG) as follows.

$DoG(x,y,\sigma)=\frac{1}{2\pi\sigma^{2}}\exp\left(-\frac{x^{2}+y^{2}}{2\sigma^{2}}\right)-\frac{1}{2\pi K^{2}\sigma^{2}}\exp\left(-\frac{x^{2}+y^{2}}{2K^{2}\sigma^{2}}\right)$ (S27)

where K = 1.6 is the ratio of two Gaussian amplitudes and $\sigma$is determined by the spatial frequency $s$ as in Equation (S6). In this paper, parvo LGN cells were modeled as having a spatial frequency $s_{p}=2.82$, whereas, the margno LGN cells has spatial frequency $s_{m}=4.57$ [12, 13]. Fig. 5c shows the radial plottings of parvo and margno DoGs in the frequency domain. To incorporate the parvo and magno systems into our model, we pre-filtered the input by convolving with two types of DoGs.

1. **Estimation of probability density functions and likelihood calculations for natural images**

It is well known that the image statistics of natural images are not Gaussian [14, 15]. Therefore, a linear template calculated from analytic expressions may not necessarily maximize proportion correct because departures from Gaussian statistics invalidate the assumption of Equation (S23). Thus, other methods are needed to estimate the template that maximizes performance.

In this paper, we estimated the statistics of natural images from the van Hateren image dataset [16], which consisted of 4212 calibrated images. We modeled the probability density function of natural images by using Laplacian distributions. First, we cropped 8 patches from 3000 natural images as a learning set, which included 24,000 image patches. Second, these patches were then correlated with a template ($\mathbf{w}=\mathbf{Va}$) built from the twenty four Gabor channels ($\mathbf{V}$) across four frequencies and six orientations.

$$r_{i}=\mathbf{w}^{T}\mathbf{g}=\mathbf{a}^{T}\mathbf{V}^{T}\mathbf{g}_{i},\text{ } i=1,...,24000$$

We found that given a set of channel weights$\mathbf{a}\in\mathbf{R}^{24}$, the distribution of these responses $\left\{r_{i}\right\}$ can be approximated well by the Laplacian distribution, as shown in Figure S2a. The probability density function of the Laplacian distribution is

$L(x)=\frac{1}{2b}\exp\left(-\frac{\left|x-\mu\right|}{b}\right)$, (S28)

where $\mu$ is a location parameter and $b>0$ is a scale parameter. Giving N independent and identically distributed samples$x_{1},\cdots x_{N}$, an ML estimator $\hat{\mu}$ of $\mu$ is the sample median [17], and the ML estimator $\hat{b}$ of b is

. (S29)

Figure S2a shows that the Laplacian distribution fits the empirical data much better than a Gaussian distribution.

We finally have to take into account the internal noise to estimate the PDF of the model. Assuming that template responses and internal noise are independent of each other, the probability density function of their sum is the convolution of the Laplacian and Gaussian distributions

$(N*L)(x)=\int_{-\infty}^{+\infty} N(u)L(x-u)du$, (S30)

where $N(x)$ and $L(x)$ are Gaussian and Laplace probability density functions respectively. Figure S2b shows an example of the three probability density functions of Gaussian, Laplacian, and the sum of these two.

7.  **Genetic Algorithm Methods**

We implemented the genetic algorithm approach to estimate the optimal linear template using the Genetic Algorithm Optimization Toolbox (GAOT) [18]. The GAOT provides a computational environment for simulated evolution in Matlab using either binary or real-valued representations. Real-valued GAs have been shown to be more efficient in finding optimal or near optimal solutions [19]. Thus, we chose a real-valued representation with roulette wheel selection, arithmetic crossover, and uniform mutation [20]. A generation consisted of 1,000 individual parameter settings, each competing on the basis of optimal task performance. For our application each individual in the population consists of two sets of twenty four weights one for each pathway. The crossover parameter was set to operate 50 times per generation, and mutation was set to operate 50 times per generation. All individuals were randomly initialized, and allowed to evolve over 500 generations. We repeated the entire GA process 10 times with different random initial values.

1. The initial population is generated randomly, then evaluated through a fitness function. In our case, the fitness is measured as the proportion correct (*Pc)* of the visual search task.
2. Genetic algorithms search the solution space of a function through the use of simulated evolution, i.e. the survival of the fittest strategy. The best or fittest individuals of any population tend to be selected and survive to the next generation, thus improving successive generations. There are several schemes for the selection process [19]. In this paper, we use roulette wheel selection [21]. This approach assigns a probability of selection, $P_{i}$, to each individual based on its fitness value: $P_{i}=\frac{Pc_{i}}{\sum_{j} Pc_{j}}$, where $Pc_{i}$ is proportion correct for the i^th^ individual. Then, a set of random numbers is generated and compared against the cumulative probability, $C_{i}=\sum_{j=1}^{i} P_{j}$. More specifically, for a uniform random variable, U(0,1), if $C_{i-1}<U(0,1)\leq C_{i}$, the individual, i, will be selected and survive to the next generation. Individuals were selected randomly with replacement until the next generation is full.
3. Before evaluating the fitness of the next generation, the processes of mutation and crossover are allowed to act on the individuals. Given two individuals, which are represented as weight vectors $\mathbf{a}=[\cdots,a_{i},\cdots]^{T}$ and $\mathbf{b}=[\cdots,b_{i},\cdots]^{T}$, we applied uniform mutation and arithmetic crossover to generate the final parameter value of the individuals, $\mathbf{a}'$and $\mathbf{b}'$. Uniform mutation randomly selected one parameter value, $a_{j}$, and sets it equal to a uniform random number U(-1,1):

$a'_{i}=\left\{\begin{matrix} U(-1,1)\text{ if }i=j \\ a_{i}\text{ if }i\neq j \end{matrix}\right.$ (S31)

Arithmetic crossover produced two complimentary linear combinations of the parents:

$\mathbf{a}'=\tau\mathbf{a}+(1-\tau)\mathbf{b}$ (S32a)

$\mathbf{b}'=(1-\tau)\mathbf{a}+\tau\mathbf{b}$ (S32b)

where $\tau=U(0,1)$ is a uniform random number.

1. The GA moves from generation to generation until a termination criterion is met. The most often used criteria include: (1) a predefined maximum number of generations; (2) no improvement of the fitness over a number of generations. We ran the GA 10 times and each run started with 1,000 random samples of channel weights, and we set the maximum number of generations to 500. This large number of generations is used to allow the algorithm to escape from local minimum.

**Supplementary References**

1. Burgess AE, Wagner RF, Jennings RJ, Barlow HB (1981) Efficiency of human visual signal discrimination. Science 214: 93–94.
2. Barrett HH (1990) Objective assessment of image quality: effects of quantum noise and object variability. J Opt Soc Am A **7:** 1266–1278.
3. Eckstein MP, Abbey CK, Bochud FO (2000) A practical guide to model observers for visual detection in synthetic and natural noisy images. In: Beutel J, Kundel HL, Van Metter RL editors. Handbook of Medical Imaging: Volume 1, Physics and Psychophysics. Bellingham: SPIE Press. pp 593–628.
4. Murray RF, Bennett PJ, Sekuler AB (2005) Classification images predict absolute efficiency. J Vision 5: 139–149.
5. Watson AB (1983) In: Bradick OJ, Sleigh AC, editors. Physical and Biological Processing of Images. New York: Springer-Verlag. pp 100–114.
6. Marcelja S (1980) Mathematical description of the responses of simple cortical cells. J Opt Soc Am A 70**:** 1297-1300.
7. Daugman JG (1985) Uncertainty relations for resolution in space, spatial frequency, and orientation optimized by two-dimensional visual cortical filters. J Opt Soc Am A 2: 1160–1169.
8. Eckstein MP, Drescher BA, Shimizaki SS (2006) Attentional cues in real scenes, saccadic targeting and Bayesian priors. Psychol Sci 17: 973–980
9. Burgess AE, Colborne B (1988) Visual signal detection. IV. Observer inconsistency. J Opt Soc Am A 5: 617-627.
10. Najemnik J, Geisler WS (2005) Optimal eye movement strategies in visual search. Nature 434: 387–391.
11. Najemnik J, Geisler WS (2009) Simple summation rule for optimal fixation selection in visual search. Vision Res 49: 1286–1294.
12. Derrington AM, Lennie P (1984) Spatial and temporal contrast sensitivities of neurones in lateral geniculate nucleus of macaque. J Physiol 357: 219-240.

# Skottun BC, Skoyles JR (2008) Spatial frequency and the magno-parvocellular distinction—some remarks. Neuro-Ophthalmology 32: 179-186.

1. Rudermant DL (1994) The statistics of natural images. Network-Comp Neural 5: 517–548.
2. Torralba A, Oliva A (2003) Statistics of natural image categories. Network-Comp Neural 14: 391–412.
3. van Hateren, JH, van der Schaaf A (1998) Independent component filters of natural images compared with simple cells in primary visual cortex. P Roy Soc Lond B Bio 265: 359–366.
4. [Norton](http://en.wikipedia.org/w/index.php?title=Robert_M._Norton&action=edit&redlink=1) RM (1984) [The double exponential distribution: using calculus to find a maximum likelihood estimator](http://www.jstor.org/pss/2683252). Am Stat 38: 135–136.
5. Houck C, Joines J, Kay M (1995) A genetic algorithm for function optimization: a Matlab implementation. NCSU-IE Technical Report 95–09.
6. Michalewicz Z (1994) Genetic Algorithms + Data Structures = Evolution Programs. New York: Springer-Verlag. 387 p.
7. [Castella C. et al (2007) Human linear template with mammographic backgrounds estimated with genetic algorithm. J Opt Soc Am A 24: 1-12.](http://www.psych.ucsb.edu/research/viu/research_pages/pdfpapers/CastellaJOSA2007.pdf)
8. Holland J (1975) Adaptation in Natural and Artificial Systems. Ann Arbor: The University of Michigan Press. 228 p.

1. The derivation is similar to Najemnik & Geisler [10] but uses the log-likelihood ratio as the decision variable rather than the posteriors. The log-likelihoods are Gaussian distributed and allow us to express the log-posteriors from previous fixations as an additive term in S24. [↑](#footnote-ref-2)
