# Supplementary material for: Evolution and Optimality of Similar Neural Mechanisms for Perception and Action during Search
Source: PLoS Comput Biol. 2010 Sep 9;6(9):e1000930. doi: 10.1371/journal.pcbi.1000930 (PMC2936525; doi:10.1371/journal.pcbi.1000930)
Supplement: Text S2 — Some notes for the manuscript. (0.03 MB DOC) [file pcbi.1000930.s004.doc]

1. Computational time (Dell Precision Workstation T7400 8 CPUs; 3.0GHz; 14GB memory)of the IS is 14.8s per trial for a single pair of perception and saccade templates which would result in 171 days for a single CPU to evaluate the 1^st^ generation with 1000 pairs of templates. We estimate that the entire virtual evolution process for the IS would take about 8716 days. In contrast, the ELM model takes about 8.9 ms per trial for a single pair of templates and 5.2 days for the entire virtual evolution.
2. Figure 1c shows that the IS fixations are somewhat more broadly distributed than the ELM model for the eight location task with broad visibility map. The current model simulations do not include saccadic landing errors present in humans. Inclusion of human random errors in the saccade endpoints in the simulations would make the ELM and IS predictions even more similar.
3. As long as the number of eye movements allowed is smaller than the number of possible target locations then guided saccades remain critical to accrue task relevant information and have an impact on the final perceptual decision performance One, three and four eye movements give similar results but due to computational demands of the simulations we used two eye movements (but see results for 8 eye movements).

Simultaneously evolving different mechanisms for different eccentricities is not computationally viable. Also, to simplify computational time we assumed an isotropic mechanism and thus equal weights across orientations.

The high frequency target is considered because one might hypothesize that a perceptual decision might be more driven by foveal processing than saccadic decisions resulting in higher frequency representation of the target for the perceptual decisions.
